# Achievement of recommended glucose and blood pressure targets in patients with type 2 diabetes and hypertension in clinical practice – study rationale and protocol of DIALOGUE

**DOI:** 10.1186/1475-2840-11-148

**Published:** 2012-12-05

**Authors:** Anselm K Gitt, Roland E Schmieder, Eva Duetting, Peter Bramlage, Steffen Schneider, Diethelm Tschöpe

**Affiliations:** 1 Stiftung Institut für Herzinfarktforschung Ludwigshafen, Bremser Strasse 79, 67063, Ludwigshafen, Germany; 2 Herzzentrum Ludwigshafen, Med. Klinik B, Ludwigshafen, Germany; 3 Universitätsklinikum Erlangen, Med. Klinik 4, Schwerpunkt Nephrologie / Hypertensiologie, Erlangen, Germany; 4 Novartis Pharma GmbH, Nürnberg, Germany; 5 Institut für Pharmakologie und präventive Medizin, Mahlow, Germany; 6 Stiftung ,Der herzkranke Diabetiker“ in der Deutschen Diabetes-Stiftung, Georgstrasse 11, 32545, Bad Oeynhausen, Germany; 7 Herz- und Diabeteszentrum Nordrhein-Westfalen in Bad Oeynhausen, Universitätsklinik der Ruhr Universität Bochum, Germany

**Keywords:** Type-2 diabetes, Hypertension, Efficacy, Effectiveness, Safety, Vildagliptin

## Abstract

**Background:**

Patients with type 2 diabetes have 2–4 times greater risk for cardiovascular morbidity and mortality than those without, and this is even further aggravated if they also suffer from hypertension. Unfortunately, less than one third of hypertensive diabetic patients meet blood pressure targets, and more than half fail to achieve target HbA1c values. Thus, appropriate blood pressure and glucose control are of utmost importance. Since treatment sometimes fails in clinical practice while clinical trials generally suggest good efficacy, data from daily clinical practice, especially with regard to the use of newly developed anti-diabetic and anti-hypertensive compounds in unselected patient populations, are essential. The DIALOGUE registry aims to close this important gap by evaluating different treatment approaches in hypertensive type 2 diabetic patients with respect to their effectiveness and tolerability and their impact on outcomes. In addition, DIALOGUE is the first registry to determine treatment success based on the new individualized treatment targets recommended by the ADA and the EASD.

**Methods:**

DIALOGUE is a prospective observational German multicentre registry and will enrol 10,000 patients with both diabetes and hypertension in up to 700 sites. After a baseline visit, further documentations are scheduled at 6, 12 and 24 months. There are two co-primary objectives referring to the most recent guidelines for the treatment of diabetes and hypertension: 1) individual HbA1c goal achievement with respect to anti-diabetic pharmacotherapy and 2) individual blood pressure goal achievement with different antihypertensive treatments. Among the secondary objectives the rate of major cardio-vascular and cerebro-vascular events (MACCE) and the rate of hospitalizations are the most important.

**Conclusion:**

The registry will be able to gain insights into the reasons for the obvious gap between the demonstrated efficacy and safety of anti-diabetic and anti-hypertensive drugs in clinical trials and their real world balance of effectiveness and safety.

## Background

There is an increasing prevalence of type-2 diabetes, which is attributable to a growing population, an increase in life expectancy, increased diagnostic efforts, and a reduced diabetes attributable risk due to recent advances in diabetes treatment [1]. Hypertension is the most frequent (90%) among the co-morbid disease conditions [2] and further increases the risk for disease and treatment related complications [3]. The combination of both hypertension and diabetes accelerates the progression of diabetes related complications such as diabetic nephropathy, retinopathy, left ventricular hypertrophy, and diastolic heart failure and doubles the risk of stroke, CV and all-cause mortality as compared to non-diabetic patients with hypertension. Risk prediction in this population is however not easy making the adaption of available risk prediction tools necessary, partly because risks have gone down in recent years [4].

### Glucose and blood pressure control

A number of studies have shown improved glycaemic control to be able to delay the onset and also to halt the progression of micro-vascular complications such as diabetic retinopathy and nephropathy. This has also been shown for neuropathic secondary disorders [5,6]. For macro-vascular complications, however, the picture to date is less straightforward. There is doubt as to whether the benefit of tight glycaemic control on macro-vascular events is as great as the one effected by blood pressure and lipid control. A recent meta-analysis of five large trials [7] found a significant reduction in event rates for non-fatal myocardial infarction and also for CAD, but all-cause mortality and stroke rates were not similarly affected.

An exploratory analysis of the United Kingdom Prospective Diabetes Study (UKPDS) data showed that the risk of both micro- and macro-vascular complications of type 2 diabetes (T2D) was strongly associated with the mean systolic blood pressure and that blood pressure lowering by 10 mmHg led to a 15 % reduction in the risk for death related to diabetes [8].

In the Steno-2 study, intensified therapy of the modifiable risk factors in patients with T2D and microalbuminuria was compared to standard treatment. In addition to lifestyle changes and diet modifications, all patients in this group received ACE-inhibitors (ACEI) or angiotensin receptor blockers (ARB) irrespective of baseline BP values and a vitamin-mineral supplement. The target limits for HbA1c, fasting cholesterol and triglycerides and blood pressure were much stricter than in the control group. This multi-factorial approach led to significant reductions in both micro- and macro-vascular event rates [9,10].

Thus, in diabetic patients with hypertension, appropriate blood pressure control as well as glucose control is important. Current guidelines [11,12] recommend a multi-factorial approach with simultaneous targeting of blood pressure and glucose levels. Unfortunately, less than one third of the hypertensive diabetics meet their blood pressure targets and less than half of them their HbA1c target.

### Pharmacotherapy

During recent years new medications and fixed dose combinations have been developed for the treatment of T2D and hypertension. For achieving glycaemic control, a number of oral therapeutic options are available, using different approaches. There are agents that increase insulin secretion, such that improve insulin action and also substances delaying carbohydrate absorption. Unfortunately, most of them – with the exception of metformin – are associated with weight gain. Hypoglycaemia and in some cases gastrointestinal side effects and oedema are other possible disadvantages. Another issue is the failure to achieve adequate control of postprandial blood glucose and also long term glycaemic control [13]. In recent years, incretin-based treatments like DPP-4 inhibitors (Vildagliptin, Sitagliptin, Saxagliptin, Linagliptin) have been shown to be a potent strategy and are increasingly used in fixed dose combinations (e.g. with metformin) [14-18]. As these drugs display different properties, e.g. pharmacokinetics, they influence the glycaemic profile in T2D – patients in different ways. This may be measure by the Mean Amplitude of Glycaemic Excursions (MAGE) and Rizzo confirmed that MAGE reduction was associated with reduction of oxidative stress and markers of systemic inflammation in T2D patients [19]. Since glucose variations over time, linked to circadian fluctuations of glucose levels, are associated with an activation of oxidative stress, the main mechanisms that lead to chronic diabetic complications [20], these data suggest that any therapy should aim not only to reduce HbA1c but also to flatten acute glucose fluctuations over time to positively influence the outcome of T2D patients.

To achieve adequate blood pressure control, an even larger number of therapy options exist. As diabetic patients frequently also have impaired renal function associated with microalbuminuria, substances affecting the renin-angiotensin system, which are known to display renal benefits independent of blood pressure reduction, appear to be particularly beneficial [21-23]. ACEI and ARB are available in several fixed dose combinations with calcium channel blockers like amlodipine and/or diuretics like hydrochlorothiazide. Interestingly, the use of fixed dose combinations has been shown to significantly improve compliance by reducing pill burden [24,25], which results in higher rates of treatment target achievement and lower hospitalisation rates.

### Guideline compliance in daily clinical practice

Treatment recommendations by current guidelines are mainly based on evidence from randomised controlled trials. However, these trials reflect only selected patient populations defined by detailed in- and exclusion criteria of these trials. Patients in daily practice are usually older and suffer from more co-morbidities as compared to those in clinical trials [26,27]. Available data suggest suboptimal treatment target achievement with respect to glucose, blood pressure and lipid control [28-31]. While there are quite some data on patient characteristics, current treatments and outcome of diabetics with hypertension in clinical practice, there are much less especially with respect to the use of newly developed and approved anti-hypertensive and anti-diabetic compounds and physicians approach towards its management [32]. Additionally, it is quite unclear if and how the different combinations of anti-diabetic and anti-hypertensive medications contribute to the achievement and preservation of target blood glucose and blood pressure values in individual patients.

### Aim

The purpose of the registry is to evaluate various therapy regimes of anti-diabetic (including incretin-based and exclusively non-incretin-based therapies) and anti-hypertensive treatments (including RAAS-inhibitors and exclusively non-RAAS-inhibitors) as well as their combinations, patient reported outcomes and treatment success in hypertensive T2D patients. It is the first prospective registry to determine treatment success based on the new individualized treatment targets of the ADA and the EASD [33].

## Methods/Design

DIALOGUE is a prospective observational national multicentre registry with a follow-up of 24 months and will enrol 10,000 patients with both T2D and hypertension from up to 700 sites in Germany. Data are recorded at baseline and will be prospectively documented during follow up visits at 6, 12 and 24 months.

This registry is conducted in accordance with the ethical principles that have their origin in the Declaration of Helsinki and adhere to the principles of Good Epidemiology Practice (GEP), and applicable regulatory requirements. The protocol of this registry was approved by the ethics committee of the Ruhr University Bochum, Germany. Patients that being enrolled into this registry will provide written informed consent. DIALOGUE has further been registered in the database of the *Verband forschender Arzneimittelhersteller* (VFA).

### Primary objective

The two co-primary objectives are: 1) documentation of individual HbA1c goal achievement with respect to anti-diabetic pharmacotherapy and 2) documentation of individual blood pressure goal achievement with different anti-hypertensive treatments.

### Secondary objective

Secondary objectives are (1) to document major cardio-vascular and cerebro-vascular events (MACCE) during 2 year follow-up; (2) to document hospitalizations during 2 year follow-up; (3) to assess the proportion of patients reaching blood glucose target values without experiencing the following adverse effects: peripheral oedema or proven hypoglycaemic events or discontinuation due to gastrointestinal events or significant weight gain (>5 %); (4) to describe patient characteristics in patients with diabetes mellitus and hypertension in clinical practice in the overall registry population; (5) to document anti-diabetic and anti-hypertensive therapy and its impact on treatment target achievements in diverse subject populations, which have to be pre-specified by the scientific committee (e.g. females versus males, age </>75y, patients on insulin versus patients not on insulin, etc.); (6) to verify the applicability of and the adherence to the current guidelines for the treatment of diabetes and hypertension in clinical practice; (7) to document utilisation patterns of drugs used for the treatment of diabetes as well as hypertension in clinical practice; (8) To evaluate adverse cardio-vascular events as well as diabetes-related micro-vascular and macro-vascular events; (9) to evaluate the glycaemic profiles of the participants with regards to differences in anti-diabetic treatment patterns; (10) to evaluate the blood pressure profiles; (11) To evaluate co-morbid disease conditions; (12) to evaluate the change in BMI over the course of the study; (13) to evaluate the proportion of patients with hypoglycaemic events over the course of the follow-up; (14) to evaluate cardio-vascular risk by using validated cardio-vascular risk scores such as the EURO Score; (15) to evaluate health status (EQ-5D); (16) to determine costs associated with the treatment and disease related complications; (17) to document treatment persistence over time, change in treatments / dosing during a follow-up of two years (optional up to 4 years of follow-up); (18) to document patient reported outcome (PRO).

### Selection of sites

The registry will be performed in primary care and diabetes centres in Germany, with a planned participation of up to 700 sites. Centres will be selected from a database maintained at the *Institut für Herzinfarktforschung* to be representative for the ambulatory treatment of diabetes and hypertension in Germany. For this purpose, a representative cross-section of different types of centres including diabetologists and primary care physicians will be built. The sampling strategy will thus provide a representative dataset for the description of oral anti-diabetic treatment patterns in Germany.

### Selection of patients

Inclusion criteria are as follows: 1) Age: ≥18 years 2) Diagnosed type 2 diabetes mellitus and manifest hypertension (comorbidity) 3) Antidiabetic therapy presently on oral mono- or dual combination therapy (no insulin, no GLP-1 analogue) 4) The treating physician considers blood glucose lowering medication to be not adequate and/or not safe/tolerable 5) The physician adds another oral drug / switches drug treatment to achieve glycaemic control 6) Written informed consent for participation obtained from the subject.

Patients will not be eligible for inclusion if any of the following exclusion criteria apply: 1.) Current participation in any randomised controlled trial. 2) Patients not under regular supervision of the treating physician for the duration of the study 3) Use of GLP-1-analogues or insulin before enrolment 4) Patients treated with aliskiren in a dual renin angiotensin aldosterone (RAAS) blockade 5) Pregnancy 6) Diabetes secondary to malnutrition, infection or surgery 7) Maturity onset diabetes of the young 8) Known cancer.

**Figure 1:**
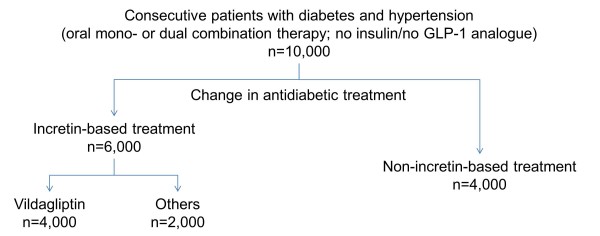
**Sample size and segmentation into strata of different antidiabetic-treatments.***Legend:* Patients are eligible for inclusion if treated with oral mono or dual combination therapy and are distributed into the following groups: Incretin-based treatments include DPP-4 inhibitors and GLP-1 analogues. Non-incretin-based therapies include metformin, sulfonylureas, acarbose, insulin, alpha-glucosidase inhibitors, and SGLT2-inhibitors.

Patients will be enrolled according to a pre-specified ratio based on their treatment, which is not pre-determined by the study protocol but based on the physician’s decision (Figure 1). “Incretin-based treatment” is defined as either a DPP-4 inhibitor or a GLP-1 analogue. “Non-incretin-based treatment” is defined as any of the following: metformin, sulfonylureas, acarbose, insulin, alpha-glucosidase inhibitors, and/or SGLT2-inhibitors. As the clinical profile of vildagliptin appears to differ from that of other DPP-4 inhibitors, the “incretin-based treatment” group will be split into those with or without vildagliptin.

### Recruitment plan

**Figure 2:**
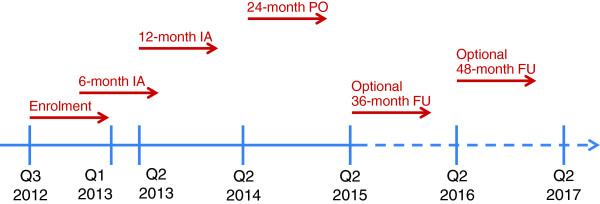
**Estimated enrolment and planned interim-analyses as well as follow-up periods.***Legend:* IA, Interim Analysis, PO, Primary Objective; FU, Follow-up.

Patient enrolment has started in July 2012. It is estimated that the first visit of the last patient enrolled will take place in early 2013, the last visit of the last patient is planned for early 2015. The Clinical Study Report will then be published in April of the following year. Figure 2 gives an overview of the timelines and the points when interim analyses to be performed.

### Documented variables

Table 1 gives an overview of the variables to be documented. Source documentation and data accuracy will be verified by site visits in randomly selected 2% of the sites.

**Table 1:** Overview of documented parameters

**Visit**	**Baseline**	**FU 6 mo**	**FU 12 mo**	**FU 24 mo**
Sociodemographics ^1^	x			
Physical examination ^2^	x			
Cardiovascular concomitant diseases ^3^	x	x	x	x
Diabetes associated diseases ^4^	x	x	x	x
Available laboratory values^5^	x	x	x	x
Antidiabetic medication^6^	x	x	x	x
Additional current medication	x	x	x	x
Hypoglycaemic events^8^	x	x	x	x
QoL (EuroQoL-5D)	x	x	x	x
Patient reported outcomes (PRO)	x	x	x	x

*Legend:* 1) age, gender, insurance status, DMP participation, education, employment status, care level 2) weight, height, waist circumference, smoking, alcohol consumption, physical activity 3) coronary heart disease, previous myocardial infarction, previous PCI, previous CABG, previous stroke, heart failure (NYHA) peripheral artery occlusive disease 4) dyslipidemia, amputation, autonomous neuropathy, non- proliferative/proliferative retinopathy, diabetic macular edema, blindness, dialysis other 5) less than six weeks old: lipid values (fasting total cholesterol, fasting LDL, fasting HDL, triglycerides), fasting and post-prandial blood glucose, HbA1c, renal values (serum creatinine, microalbustix albumin, microalbustix creatinine), liver parameters. 6) metformin, sufonylureas, glucosidase inhibitors, glinides, glitazones, DPP-4 inhibitors) 7) ACE inhibitors, ARBs, renin inhibitors, beta blockers, calcium channel blockers, diuretics, other) 8) 12 months before baseline visit and since baseline or last FU, respectively.

### Quality assurance

There are three strategies for data quality checks: validations that occur at the time of data entry (i.e., “front-end”), a second, more sophisticated quality control program that runs as a prelude to the creation of the analysis data set and on-site data monitoring.

Front-end data checks are advantageous because mistakes are caught and corrected at the time of entry – a system that is efficient for data collectors. Certain data elements can be required, while other variables may allow for missing values. Additionally, parameters will be defined to allow entry of only those records that meet inclusion criteria.

Prior to the creation of the analytic dataset, more extensive quality control processes are performed. These checks, programmed in SAS, include parent–child edits, consistency edits, and data transformations that will facilitate analyses.

Source documentation and data accuracy will be verified by site visits in randomly selected 2% of the sites.

### Statistical methods

All variables collected in the eCRF as well as the data obtained from the quality of life assessments and all derived parameters will be used in the statistical analysis. Binary, categorical, and ordinal parameters will be summarised by means of absolute and percentage numbers within the various categories (including ‘missing data’ as valid category at baseline). Numerical data will be summarised by means of standard statistics (i.e. number of available data, number of missing data, mean, standard deviation, minimum, median, maximum, lower and upper quartile). In addition, adequate graphs (e.g. bar charts, box-whisker plots) may be presented to summarise the results for some parameters. Time-to-event variables will be analysed via a Cox proportional hazard regression model presenting hazard ratios and the corresponding 95% confidence intervals. In addition Kaplan-Meier curves will be presented for these variables. Two-sided 95%-CI will be presented for important parameters, but should be interpreted in an exploratory descriptive way. Further multivariable analyses will be performed according to the statistical analysis plan (SAP). Formal statistical tests will not be performed within the statistical analysis. A report including descriptive statistics of all documented parameters will be generated for the overall patient population. Depending on the variable(s) of interest, additional selection criteria for patients (e.g. subgroup analyses) considered in specific analyses may be used, if considered useful during the statistical analysis. Details on the selection criteria used will be given in the SAP and in the statistical section of the report. The statistical analysis will be performed using SAS (release 9.2 or higher; Cary, NC, USA).

## Discussion

There are a number of epidemiological studies, which documented (among other aspects) the treatment of type-2 diabetes in Germany. Table 2 gives an overview of those studies closely related to the objectives of DIALOGUE. These include DUTY [34], DIG [35-37] and DiaRegis [2,38-40]. DUTY (2001–2003) was among the first [34] to prospectively document the effect of a tailored intervention on blood glucose and CV risk factor target achievement. They demonstrated that too many patients suffering from diabetes mellitus do not receive consistent therapy for cardiovascular risk factors according to guidelines and therefore the required target values were rarely reached. DIG [35-37] started one year later (2002) and had a four year follow-up and meant to document guideline-oriented treatment across Germany. A major secondary focus was to investigate the metabolic syndrome in Germany. DiaRegis [2,38-40] is a prospective registry focussing on the role of hypoglycaemia on subsequent vascular events. Throughout the follow-up a steady increase in the incidence of vascular events was documented, suggesting an association between hyperglycaemia and vascular events [41].

**Table 2:** Comparison of DIALOGUE with other existing registries

	**DIALOGUE**	**DiaRegis**	**DIG**	**DUTY**
Reference		[2,38-40]	[35-37]	[34]
No. of physicians	Up to 700	313	238	n.a.
No. of patients	Up to 10,000	3,810	4,020	59,035
Recruitment	Starting 06/2012	06/2009–03/2010	2002-2004	2001-2003
Follow-up	2-4 years	2 years	4 years	9 months
Design	Prospective cohort	Prospective cohort	Prospective cohort study	Prospective cohort study
Monitoring for data verification	Yes (2%)	Yes (10%)	None	None
Proportion T2D	100%	100%	100%	100%
Patients	Co-morbid disease of diabetes and hypertension	Patients on oral mono- or dual antidiabetic combination therapy	Type-2 Diabetes mellitus	Type-1 or type-2 diabetes mellitus
Median age (years)	n.a.	65.9	Mean 61.8 ± 8.1	64.4 ± 11.7
Female (%)	n.a.	46.7	46.8	50.9
BMI (median)	n.a.	30.0	Mean 30.7 ± 5.2	Mean 28.7 ± 4.8
Focus	Target achievement with respect to HbA1c and blood pressure	Hypoglycaemia incidence with antidiabetic drug use	Application of guidelines in clinical practice	Effect of tailored intervention on target achievement

*Legend.* n.a., not available.

Most of these studies however either had a detailed look on the prevalence of type-2 diabetes in primary care practice (HYDRA and DETECT), the co-morbidity burden (DETECT), the costs (CODE-2, CoDiM and ROSSO), or on self-monitoring of blood glucose (ROSSO). Some were also retrospective in design (CODE-2, CoDiM and ROSSO). Similar to DIALOGUE the effect of a tailored intervention on target achievement was investigated in DUTY and DIG. The added value of DIALOGUE is, that it will enrol almost 10,000 patients exclusively with the co-morbid disease constellation diabetes *and* hypertension and the effect of two matching treatment approaches on target goal achievement as to the more recent guidelines which suggest more individualized treatment targets [33,42,43] based on increasing calls for a move toward more patient-centred care [44,45].

## Conclusions

DIALOGUE is the first registry that focuses at the evaluation of T2D patients who also suffer from hypertension, addressing both achievement of glycaemic and anti-hypertensive goals considering the individualized treatment targets as recommended by recent guidelines. As the combination of both diseases is a major factor in the development of vascular complications, data obtained will help to explain the gap between the inadequate glycaemic and blood pressure control in the “real world setting” despite the demonstrated efficacy and safety of anti-diabetic and anti-hypertensive drugs in clinical trials.

## Abbreviations

MACCE: Major Cardiovascular and Cerebrovascular Events; MAGE: Mean Amplitude of Glycaemic Excursions; T2D: Type-2 Diabetes Mellitus; UKPDS: United Kingdom Prospective Diabetes Study; ACEI: ACE-inhibitor; ARB: Antiotensin Receptor Blocker; RAAS: Renin Angiotensin Aldosterone System.

## Competing interests

Anselm K Gitt (AKG), Roland E. Schmieder (RES), Peter Bramlage (PB), and Diethelm Tschöpe (DT) have received research support and honoraria for lectures from a number of pharmaceutical companies producing anti-diabetic drugs including Novartis the sponsor of this study. Eve Duetting (ED) is employee of the sponsor. Steffen Schneider (StS) has no potential conflict of interest to disclose.

## Authors’ contributions

AKG, ED, RES and DT designed the registry. StS is responsible for the analysis of data. PB drafted the manuscript based on the protocol and all other authors revised the article for important intellectual content. All authors have finally approved the version to be published.

## Authors’ information

DIALOGUE study group: Sibel Avsar (Ludwigshafen), Peter Bramlage (Mahlow), Eva Duetting (Nürnberg), Anselm K. Gitt (Ludwigshafen), Alexander Neumer (Ludwigshafen), Taoufik Ouarrak (Ludwigshafen), Roland E. Schmieder (Erlangen), Steffen Schneider (Ludwigshafen), Diethelm Tschöpe (Bad Oeynhausen).
